# Patterns of Changes in Respiratory Muscle Strength over 1 Year in Non-Sarcopenia, Sarcopenia, and Severe Sarcopenia

**DOI:** 10.3390/ijerph192416571

**Published:** 2022-12-09

**Authors:** Yohei Sawaya, Tamaki Hirose, Masahiro Ishizaka, Takahiro Shiba, Ryo Sato, Akira Kubo, Tomohiko Urano

**Affiliations:** 1 Department of Physical Therapy, School of Health Sciences, International University of Health and Welfare, 2600-1 Kitakanemaru, Otawara 324-8501, Tochigi, Japan; 2 Nishinasuno General Home Care Center, Department of Day Rehabilitation, Care Facility for the Elderly “Maronie-en,” 533-11 Iguchi, Nasushiobara 329-2763, Tochigi, Japan; 3 Department of Geriatric Medicine, School of Medicine, International University of Health and Welfare, 4-3 Kozunomori, Narita 286-8686, Chiba, Japan

**Keywords:** elderly, sarcopenia, respiratory sarcopenia, Japan, maximum expiratory pressure

## Abstract

In this prospective longitudinal cohort study, we explored the characteristics of older people with lower respiratory muscle strength, according to sarcopenia severity, over the course of 1 year. The maximum expiratory pressure (MEP), grip strength, walking speed, and skeletal muscle mass index of 58 participants (28 men, 30 women; mean age, 76.9 ± 7.7 years) were measured at baseline and at the 1-year follow-up. Participants were classified into a decreased MEP group (*n* = 29; MEP decreased by ≥10% after 1 year) and a non-decreased MEP group (*n* = 29; MEP decreased by <10%). Sarcopenia status in the mild direction at baseline was significantly associated with MEP decline after one year. Repeated two-way analysis of variance showed significant main effects of measurement time (*p* < 0.001) and severity of sarcopenia (*p* = 0.026), as well as a significant interaction effect (*p* = 0.006). Surprisingly, MEP decreased significantly in the non-sarcopenia and sarcopenia groups, but not in the severe sarcopenia group. Thus, individuals without sarcopenia and those with moderate sarcopenia at baseline are predisposed to MEP decline and should be closely monitored for signs of such decline and associated adverse events.

## 1. Introduction

The concept of respiratory sarcopenia was first proposed in 2021, and it has since been defined as “whole-body sarcopenia and low respiratory muscle mass followed by low respiratory muscle strength and/or low respiratory function” [1]. Respiratory sarcopenia can be caused by various factors, such as ageing, reduced activity levels, undernutrition, hypoxic stress, disease, and cachexia [1,2]. Furthermore, respiratory muscle mass and strength also decrease with age, much like all other muscles of the body [1,3].

Respiratory sarcopenia is diagnosed based on the evaluation of respiratory muscle mass and strength. However, measuring respiratory muscle mass can often be complicated because it requires advanced diagnostic equipment or techniques, such as ultrasound echography [4,5,6] and computed tomography [7,8]. Respiratory muscle strength is simpler to evaluate because it can be measured with spirometry; however, a consensus is yet to be reached on the exact methodology. For example, there are no established cut-offs for maximal inspiratory pressure (MIP) or forced vital capacity, as proposed by Nagano et al. [1]. Kera et al.—who first reported respiratory sarcopenia—also proposed peak expiratory flow rate (PEFR) as the main indicator of this disease [9]. The results of our previous study indicated that maximal expiratory pressure (MEP, which represents expiratory muscle strength) is more closely associated with whole-body sarcopenia than maximal inspiratory pressure (MIP, which represents inspiratory muscle strength) [10].

Previous studies on respiratory sarcopenia have reported a decrease in respiratory muscle strength and respiratory function among older adults with sarcopenia [9,10,11,12]. Older adults with sarcopenia are reported to have a thin diaphragm [13]. Furthermore, there is a difference in physical function and muscle strength between people with lower respiratory muscle strength associated with sarcopenia and those with lower respiratory muscle strength alone [14]. However, no previous studies have investigated the changes in this relationship over time because previous studies on respiratory sarcopenia have primarily used cross-sectional study designs.

Although it is clear that respiratory sarcopenia is associated with negative outcomes, elucidating the characteristic of this association by accumulating further evidence is essential. In this study, we evaluated changes in respiratory muscle strength over a 1-year period in participants classified by severity of sarcopenia. In addition, we aimed to identify the baseline characteristics of individuals who experienced a decrease in respiratory muscle strength. Our results help elucidate the changes in respiratory muscle strength over time considering the concept of respiratory sarcopenia. We hypothesized that MEP decline would positively correlate with the severity of sarcopenia.

## 2. Materials and Methods

### 2.1. Study Design

This single-center 1-year prospective cohort study was conducted from March 2018 to August 2019, and it is a follow-up study to our previous cross-sectional study [10]. The study was explained to all participants orally and in writing, and each participant provided their written informed consent. This study was approved by the ethics committee of the International University of Health and Welfare (approval no.: 17-Io-189-7) and was conducted in accordance with the Declaration of Helsinki.

### 2.2. Study Participants

**Figure 1:**
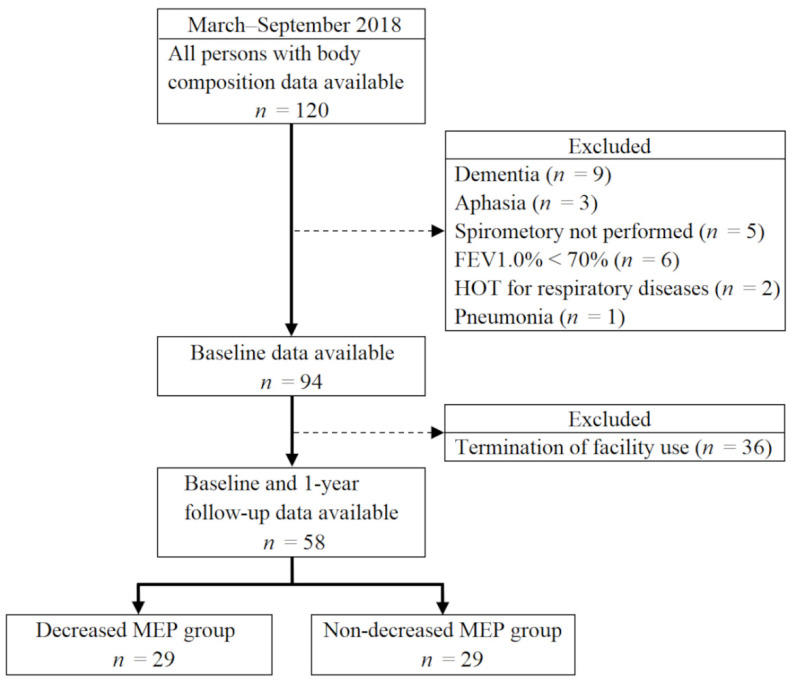
Flowchart of participant selection. FEV1.0%, forced expiratory volume % in one second; HOT; home oxygen therapy; MEP, maximum expiratory pressure.

The participants were community-dwelling older adults aged ≥60 years who used a day-care service for older adults [15]. This is a day-care with rehabilitation service (including transportation services) that operates under the Japanese Long-term Care Insurance system. Participants were recruited from 120 individuals who met the inclusion criteria of being able to have their body composition measured in the standing position during the baseline period of March–August 2018. Individuals who met the following criteria were excluded: difficulty in understanding instructions for the measurement of respiratory muscle strength due to dementia or aphasia at baseline, difficulty in measuring respiratory muscle strength, FEV1.0% < 70%, respiratory disease requiring home oxygen therapy (such as chronic obstructive pulmonary disease), and a history of pneumonia. Baseline data were available for 94 individuals; of these, 36 were excluded because they had discontinued their usage of the day-care service at the time of the 1-year follow-up. Finally, the data for 58 individuals (28 men, 30 women; mean age, 76.9 ± 7.7 years) were ultimately included in the analysis (Figure 1).

### 2.3. Classification by Severity of Reduction in Respiratory Muscle Strength

We used the extent of decrease in MEP as an indicator of the reduction in respiratory muscle strength. Participants whose MEP decreased by ≥10% between baseline and the 1-year follow-up were classified into the decreased MEP group (*n* = 29), and those with a <10% decline in MEP were classified into the non-decreased MEP group (*n* = 29).

### 2.4. Respiratory Muscle Strength

A spirometer (Autospiro AS-507, Minato Medical Science, Osaka, Japan) and its attachment (AAM377, Minato Medical Science, Osaka, Japan) were used to evaluate the MEP and PEFR values of the participants. All measurements were recorded by a physiotherapist using the American Thoracic Society/European Respiratory Society guidelines as a reference [16]. The PEFR values were recorded first, followed by the MEP values. All measurements were repeated three times, and the maximum of the three trials was recorded for analysis. All measurements were recorded with participants in the sitting position.

We used two models to assess the reduction in baseline respiratory muscle strength: the Sawaya model, which uses the MEP (men, 47.0 cmH_2_O; women, 40.9 cmH_2_O); and the Kera model, which uses the PEFR (men, 4.40 m/s; women, 3.21 m/s) [9,10]. Participants who performed below the reference value for respiratory muscle strength in each model were classified as having lower respiratory muscle strength at baseline.

### 2.5. Sarcopenia Assessment

Sarcopenia was diagnosed taking sex into account according to the guidelines of the Asian Working Group for Sarcopenia 2019 and was classified into three levels of severity: non-sarcopenia, sarcopenia, and severe sarcopenia [17]. Sarcopenia corresponded to low skeletal muscle mass in combination with either low muscle strength or low physical function, whereas severe sarcopenia corresponded to the presence of all three conditions: low skeletal muscle mass, low muscle strength, and low physical function [17]. Grip strength was measured in the sitting position using the Smedley hand dynamometer (TKK 5401 Grip-D, Takei Scientific Instruments, Niigata, Japan) twice for each hand, and the higher value of the two trials was recorded for analysis. Normal walking speed was measured once with a stopwatch on a path (length, 5 m) with a segment for acceleration and deceleration [18]. Skeletal muscle mass was measured using a multifrequency bioelectrical impedance analysis (BIA) device (InBody 520, InBody, Japan). The skeletal muscle mass index (SMI) was calculated as the skeletal muscle mass of the four limbs divided by the height squared.

### 2.6. Basic Attributes

Data regarding the participants’ age, height, long-term care certification level, smoking history, and disease history were collected from the patient medical records. The Japanese long-term care system classifies the independence of older adults on a 7-point ordinal scale (1: relatively independent older adults needing only minimal assistance; 7: older adults requiring the most amount of assistance) [15].

### 2.7. Statistical Analysis

The decreased MEP and non-decreased MEP groups were compared using the unpaired *t*-test, the Mann–Whitney *U* test, the chi-square test, and Fisher’s exact test. Binary logistic regression analysis was performed with the following conditions: the presence or absence of MEP decrease was the dependent variable, and the severity of sarcopenia and presence or absence of respiratory muscle strength decline at baseline were independent variables. This multivariate analysis was performed to investigate which of the following variables was associated with a reduction in respiratory muscle strength after 1 year: (1) severity of sarcopenia at baseline or (2) lower respiratory muscle strength at baseline, assessed using the Sawaya and Kera models, which clearly define the cut-off value for Japanese individuals [9,10]. In addition, we compared MEP values using a repeated two-way analysis of variance (ANOVA) with measurement timing (baseline/follow-up) and severity of sarcopenia at baseline (non-sarcopenia, sarcopenia, severe sarcopenia) as the two factors. Factors for which the main effect and interaction effect were significant were further analyzed with a simple main effect test.

Statistical analysis was performed using IBM SPSS version 25 (IBM Japan, Tokyo, Japan) and G*Power version 3.1.9.2. The significance level was set at 5%. The paired *t*-test, unpaired *t*-test, and repeated two-way ANOVA were performed after confirming the normal distribution of the variables with the Kolmogorov–Smirnov test/Shapiro–Wilk test.

## 3. Results

Table 1 lists the baseline characteristics of the participants in the decreased and non-decreased MEP groups. The severity of sarcopenia, SMI, and MEP were significantly different between the groups. Binary logistic regression analysis showed that the decline in MEP was associated with sarcopenia status in the mild direction at baseline (Table 2). The results of the post hoc power analysis were 0.99 and 0.98 for models I and II, respectively. The Kolmogorov–Smirnov test and the Shapiro–Wilk test were used to confirm the normal distribution of residuals of both models.

**Figure 2:**
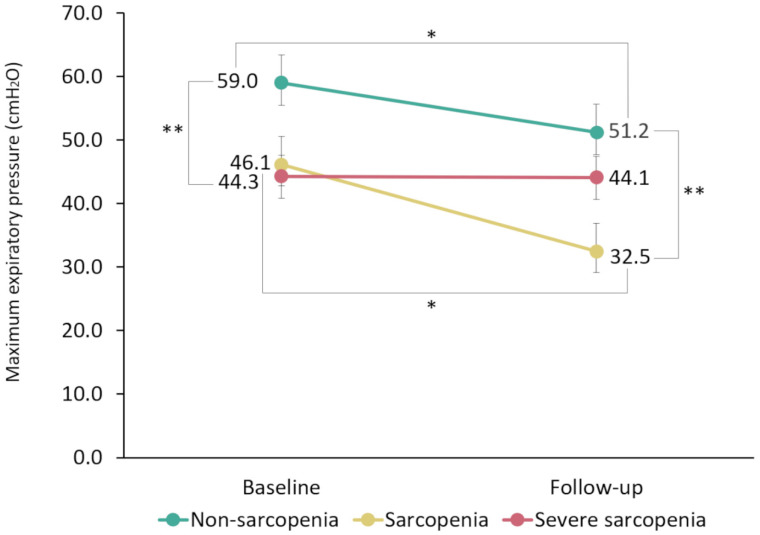
One-year changes in maximum expiratory pressure (MEP) in the non-sarcopenia, sarcopenia, and severe sarcopenia groups based on repeated two-way analysis of variance. Bars in the figure indicate the standard errors. MEP shows a significant interaction between the measurement time and severity of sarcopenia. Measurement time (baseline, follow-up) and severity of sarcopenia (non-sarcopenia, sarcopenia, severe sarcopenia) had significant main effects on MEP values. * Significant simple main effect of measurement time. ** Significant multiple comparison of sarcopenia severity (significant simple main effect observed at both baseline and follow-up).

Figure 2 shows the changes in MEP over a 1-year period in the non-sarcopenia, sarcopenia, and severe sarcopenia groups. A repeated two-way ANOVA showed significant main effects of the measurement time (F = 17.9, *p* < 0.001) and severity of sarcopenia (F = 3.9, *p* = 0.026), as well as a significant interaction effect (F = 5.6, *p* = 0.006). Duration had a significant simple main effect on MEP decline in non-sarcopenia and sarcopenia for term. As for factors of the severity of sarcopenia, a significant simple main effect was observed at baseline and follow-up, and multiple comparison analysis revealed significant differences between baseline non-sarcopenia and severe sarcopenia and between non-sarcopenia and sarcopenia at follow-up.

## 4. Discussion

Respiratory sarcopenia is a recent concept, and the definition for this condition was first proposed in 2021 [1]. Therefore, experts have yet to reach a consensus on its definition, and the validity and reliability of the diagnostic criteria have not yet been evaluated in detail. Furthermore, most of the studies on respiratory sarcopenia to date have consisted of cross-sectional designs [9,10,11,12,13,14], and the patient characteristics that predict a decline in respiratory muscle strength have not been identified. This study is the first to report changes in respiratory muscle strength over time according to the severity of sarcopenia. Thus, although our results are based on a small sample size, they still provide valuable new evidence to elucidate the pathogenesis of respiratory sarcopenia.

In this 1-year longitudinal study, MEP decreased significantly in the non-sarcopenia and sarcopenia groups, although there were no significant changes in MEP among individuals with severe sarcopenia, contrary to our hypothesis. The multivariate analysis revealed that sarcopenia status in the mild direction as a baseline factor was associated with a decline in MEP. The rate of MEP decline was the highest in the sarcopenia group. This suggests that people without sarcopenia and those with sarcopenia (but not severe sarcopenia) are predisposed to a gradual decline in MEP. Therefore, it is important to monitor patients for signs of MEP decline and associated adverse events, even in older adults who appear to be in good physical function with good muscle strength/moderate sarcopenia. Aspiration pneumonia is a typical adverse event associated with a decline in respiratory muscle strength and respiratory function, and it is a common cause of death in older adults [19]. Several studies have reported that respiratory decline is a risk factor for aspiration pneumonia [20,21,22]. In addition, other studies have reported a vicious cycle of sarcopenia progression following pneumonia [23,24]. A reduction in respiratory muscle strength is also associated with a decline in mobility, even after controlling for several covariates [25]. This suggests that interventions aimed at improving respiratory muscle strength may potentially decrease adverse events in older adults.

Morisawa et al. noted lower respiratory muscle strength in approximately 50% of patients without whole-body sarcopenia, suggesting that lower respiratory muscle strength may be a precursor to sarcopenia [14]. The underlying physiology of this disease may be explained by the notion of respiratory muscle-induced metaboreflex. This theory suggests that a decline in respiratory muscle mass and strength precedes a reduction in exercise tolerance, primarily due to reduced oxygen carrying capacity, skeletal muscle fatigue in the extremities, and general muscle weakness [14,26,27,28].

The results of our repeated two-way ANOVA indicated that patterns of changes in MEP differed between the groups depending on the severity of sarcopenia. In particular, the gradual age-related changes in MEP suggest that the decrease in MEP may stop in patients with severe sarcopenia [29]. However, future studies are needed to determine whether the respiratory muscles will reach a sort of default minimal muscle mass.

There are several limitations to this study. First, because the sample size was small, our analyses could not be stratified by sex. However, the male-to-female ratio was almost equal (both in the entire study population and in the groups divided by severity of sarcopenia). There was also a large number of drop-outs at the 1-year follow-up. Second, the definition of MEP decrease (≥10% in 1 year) used in this study may be arbitrary. Nonetheless, this cut-off value divided the sample into two approximately equal-sized groups. Third, the data for participants in this study may have been affected by the exercise program offered at the day-care service for older adults. As we included older Japanese adults with multiple comorbidities requiring long-term care, we could not eliminate all disease-related factors.

## 5. Conclusions

Our results revealed different patterns of changes in MEP depending on the severity of sarcopenia. Individuals with non-sarcopenia and sarcopenia were predisposed to experiencing a reduction in respiratory muscle strength. Therefore, it is important to closely monitor older adults for a decrease in MEP and associated adverse events, including older adults without sarcopenia or those with moderate sarcopenia.

## Figures and Tables

**Table 1:** Basic attributes and measurements at baseline.

	Decreased MEP Group (*n* = 29)	Non-Decreased MEP Group (*n* = 29)	*p*-Value
Men/Women	15/14	13/16	0.793
Age (years)	77.9 ± 8.0	75.9 ± 7.3	0.335
Height (cm)	158.2 ± 6.8	156.4 ± 7.6	0.330
Weight (kg)	57.2 ± 9.5	55.0 ± 9.2	0.367
Body mass index (kg/m^2^)	22.8 ± 3.2	22.5 ± 3.3	0.676
Certification level (1–7) ^†^	3 (1.5–4)	3 (1–3.5)	0.896
Smoking history	10	7	0.565
Sarcopenia assessment			
Non-sarcopenia	16	7	0.003 *
Sarcopenia	6	2	
Severe sarcopenia	7	20	
Grip strength (kg)	23.1 ± 6.7	20.3 ± 8.2	0.153
Normal walking (m/s)	0.69 ± 0.33	0.55 ± 0.26	0.095
SMI (kg/m^2^)	6.27 ± 0.84	5.78 ± 0.95	0.042 *
Respiratory assessment			
Low RMS Sawaya model	5	16	0.006 *
Low RMS Kera model	17	16	1.000
MEP (cmH_2_O)	57.7 ± 20.9	43.1 ± 14.7	0.003 *
PEFR (L/s)	3.45 ± 1.43	3.80 ± 1.45	0.353
Morbidity			
Cerebrovascular disease	17	14	0.599
Orthopedic disease	12	18	0.189

SMI, skeletal muscle mass index; RMS, respiratory muscle strength; MEP, maximum expiratory pressure; PEFR, peak expiratory flow rate. Decreased respiratory muscle strength = the presence or absence of a ≥10% decrease in MEP over a period of 1 year. * *p*-value set to 0.05. † Median (25th–75th percentile).

**Table 2:** Results of a binomial logistic regression analysis to investigate associations between decreased MEP and baseline assessments of sarcopenia and respiration.

**Model I**	**β**	***p*-Value**	**Odds Ratio**	**95%CI**
Sarcopenia diagnosis ^†^	−0.879	0.023 *	0.415	0.194–0.887
Low RMS Sawaya model ^††^	−1.568	0.054	0.209	0.042–1.027
**Model II**	**β**	***p*-Value**	**Odds Ratio**	**95%CI**
Sarcopenia diagnosis ^†^	−1.239	0.001 *	0.290	0.138–0.609
Low RMS Kera model ^††^	0.550	0.395	1.734	0.487–6.168

MEP, maximum expiratory pressure; RMS, respiratory muscle strength; CI, confidence interval. Dependent variables: non-decreased MEP = 0, ≥10% decreased MEP = 1. Independent variables: † Non-sarcopenia = 0, sarcopenia = 1, severe sarcopenia = 2; †† Non-low RMS = 0, low RMS = 1. Controlling for sex, age, body mass index, and cerebrovascular disease in both models. * *p*-value set to 0.05.

## Data Availability

Research data are not shared.
